# Pediatric inherited peripheral neuropathy: a prospective study at a Spanish referral center

**DOI:** 10.1002/acn3.51432

**Published:** 2021-07-29

**Authors:** Herminia Argente‐Escrig, Marina Frasquet, Juan Francisco Vázquez‐Costa, Elvira Millet‐Sancho, Inmaculada Pitarch, Miguel Tomás‐Vila, Carmen Espinós, Vincenzo Lupo, Teresa Sevilla

**Affiliations:** ^1^ Neuromuscular & Ataxias Research Group Instituto de Investigación Sanitaria La Fe Valencia Spain; ^2^ Neuromuscular Diseases Unit Department of Neurology Hospital Universitari i Politècnic La Fe Valencia Spain; ^3^ Centre for Biomedical Network Research on Rare Diseases‐CIBERER Valencia Spain; ^4^ Rare Diseases Joint Unit IIS La Fe – CIPF Valencia Spain; ^5^ Department of Clinical Neurophysiology Hospital Universitari i Politècnic La Fe Valencia Spain; ^6^ Department of Pediatrics Neuropediatrics Unit Hospital Universitari i Politècnic La Fe Valencia Spain; ^7^ Unit of Genetics and Genomics of Neuromuscular and Neurodegenerative Disorders Centro de Investigación Príncipe Felipe (CIPF) Valencia Spain; ^8^ Department of Medicine University of Valencia School of Medicine Valencia Spain

## Abstract

**Background:**

Single‐center clinical series provide important information on genetic distribution that can guide genetic testing. However, there are few such studies on pediatric populations with inherited peripheral neuropathies (IPNs).

**Methods:**

Thorough genetic testing was performed on IPN patients under 20 years of age from a geographically well‐defined Mediterranean area (Valencian Community, Spain), annually assessed with the Charcot–Marie–Tooth disease Pediatric Scale (CMTPedS).

**Results:**

From 86 families with IPNs, 99 patients (59 males) were identified, 85 with sensorimotor neuropathy or CMT (2/3 demyelinating form) and 14 with distal hereditary motor neuropathy (dHMN). Genetic diagnosis was achieved in 79.5% families, with a similar mutation detection rate in the demyelinating (88.7%) and axonal (89.5%) forms, significantly higher than in the dHMN families (27.3%). CMT1A was the most common subtype, followed by those carrying heterozygous mutations in either the *GDAP1* or *GJB1* genes. Mutations in 15 other genes were identified, including a new pathogenic variant in the *ATP1A* gene. The CMTPedS detected significant disease progression in all genetic subtypes of CMT, at a rate of 1.84 (±3.7) over 1 year (*p* < 0.0005, *n* = 62) and a 2‐year rate of 3.6 (±4.4: *p* < 0.0005, *n* = 45). Significant disease worsening was also detected for CMT1A over 1 (1.7 ± 3.6, *p* < 0.05) and 2 years (4.2 ± 4.3, *p* < 0.0005).

**Conclusions:**

This study highlights the unique spectrum of IPN gene frequencies among pediatric patients in this specific geographic region, identifying the CMTPedS as a sensitive tool to detect significant disease worsening over 1 year that could help optimize the design of clinical trials.

## Introduction

Inherited peripheral neuropathies (IPNs) are a complex group of diseases with broad phenotypic and genotypic diversity, especially in pediatric populations. Charcot–Marie–Tooth disease (CMT) represents a group of inherited neuropathies with both motor and sensory involvement, and it is generally classified according to the upper limb motor nerve conduction velocities (MNCVs): demyelinating CMT1 when the MNCV <38 m/s; or axonal CMT2 if the MNCV >38 m/s.^1^ In some cases, the term “intermediate CMT” is also used when upper limb MNCVs are between 35 m/s and 45 m/s.^2^ The motor‐predominant and sensory‐predominant ends of the spectrum are referred to as distal hereditary motor neuropathy (dHMN) and hereditary sensory neuropathy (HSN), respectively.

Advances in molecular genetics in the past 30 years have provided an ever‐expending list of more than 90 genes and loci implicated in IPNs, especially after the discovery of next‐generation sequencing (NGS).^3^ Characterizing large series of patients clinically and genetically is important to obtain information about the natural history and the phenotype‐gene relationships in IPNs. In the last 5 years, the multi‐center joint effort of the Inherited Neuropathies Consortium (INC) determined the genetic spectrum of CMT in their entire cohort,^4^ as well as in the subgroup aged 3–20 years old.^5^

Nevertheless, analyzing clinical series from single‐centers in a uniform and comprehensive manner is still important to shed light on the genetic distributions in these populations, and in geographically well‐defined areas, helping to guide genetic testing. The significant delay in diagnosing pediatric IPN populations^6^ and the absence of widespread validated tools to measure disability in this age group until recently^7^ has led to a paucity of large single‐center cohorts of children with IPNs^8^, ^9^ relative to adult cohorts.^2^, ^10^, ^11^, ^12^, ^13^ Here we report the genetic distribution, phenotypic characterization and natural history over 2 years of disease progression in an extensive series of pediatric IPNs from a single referral center located in a Spanish region with 5.000.000 inhabitants (Valencian Community).

## Methods

### Patients

This is a longitudinal descriptive study carried out on all patients in whom IPN was the leading feature and who were evaluated prior to the age of 20 at the Neuromuscular Clinic of the Hospital Universitari i Politècnic La Fe (Valencia, Spain), between 2017 and 2020. The diagnosis and classification of IPNs was based on the clinical manifestations, family history, and electrophysiological features.^14^ According to sensory nerve conduction studies (NCSs), patients were classified as having CMT (if the sensory NCS was abnormal) or dHMN (if normal). Patients were subclassified as having demyelinating or axonal CMT based on their forearm ulnar MNCV, with a cut‐off value of 38 m/s.^1^ In patients whose amplitudes of ulnar compound motor action potentials (CMAPs) were reduced >90%, we considered the conduction velocities measured to the flexor carpi ulnaris or the axillary latency.

### Nerve conduction studies

NCSs were performed by standard techniques using a Medelec Synergy electromyograph (Mistro, Surrey, UK), with surface electrode stimulation and recording. Recently published normal values were employed.^15^ Electrophysiological recordings were taken from the motor ulnar, median, axillary, peroneal nerves and the sensory (orthodromic) median, (antidromic) sural, and radial nerves. Distal motor latency (DML), MNCV, sensory nerve conduction velocity (SNCV), and the amplitudes (baseline to negative peak) of CMAPs and sensory action potentials (SAPs) were also assessed.

Written informed consent was obtained from the patients themselves or their guardians. This study was approved by the Institutional Review Board of Hospital Universitari i Politècnic La Fe.

### Clinical assessments and disease severity

As clinical features of the subjects, we assessed their strength, muscular atrophy, sensory responses, reflexes, and foot deformities, and we conducted a general and a neurological examination. Foot deformity was assessed using the Foot Posture Index,^16^ while the ankle joint dorsiflexion was measured by weight bearing using the lunge test and a bubble inclinometer. No achilles retraction was present if the lunge test >35°.^17^ The Charcot–Marie–Tooth disease Pediatric Scale (CMTPedS) was administered by the same examiner (HAE) to quantify disease severity in a subset of patients, at baseline, and after 1 and 2 years of disease progression. We were unable to assess the CMTPedS in some patients with intellectual disability or behavioral issues that limited their collaboration. Using the online CMTPedS calculator (https://www.cmtpeds.org), the 11 performance‐based items of dexterity, strength, sensation, balance, gait, power, and endurance were converted to categorized scores, ranging from 0 (unaffected) to 4 (severely affected), and these scores were summed to produce a total CMTPedS score ranging from 0 to 44, whereby a higher score indicates greater disease severity. A score of 0–14 is considered mildly affected, a score of 15–29 is defined as moderate, and 30 points or above is considered as severe.^7^

### Molecular genetic analysis

Sanger sequencing on an ABI Prism 3730xl (Applied Biosystems, Foster City, CA, USA) was performed to identify the disease‐causing mutation in genetically diagnosed families. In all of the probands with demyelinating CMT, the CMT1A duplication was first analyzed by MLPA (Multiplex Ligation‐dependent Probe Amplification) on a genetic analyzer ABI Prism 3130xl (Applied Biosystems, Foster City, CA, USA) and using the CMT1 SALSA kit P033‐B4 (MRC‐Holland, Amsterdam, the Netherlands). If a CMT1A duplication was ruled out, mutational screening of the *SH3TC2*, *NDRG1,* and *HK1* genes was performed in demyelinating CMT patients of the Roma minority, as described previously.^18^ In the remaining undiagnosed index patients, an NGS‐targeted custom panel was employed using the Sure Select QXT technology of Agilent technologies (Santa Clara, CA, USA). Any genetically undiagnosed patients after assessment with the gene panel subsequently underwent whole exome sequencing. In all cases, NGS libraries were sequenced using an Illumina system (San Diego, CA, USA). After variant annotation and filtering, variant classification was carried out based on the American College of Medical Genetics (ACMG) guidelines.^19^ All pathogenic variants were validated by Sanger sequencing.

### Statistical analysis

Statistical analysis was performed with SPSS v. 20.0 (IBM Corp. Armonk, NY). Descriptive data were represented as the mean ± standard deviation (SD) or as percentages. All data were assessed for normality and the appropriate parametric or nonparametric tests were subsequently used. We used paired *t*‐tests to assess the significance of change in the CMTPedS total score between the baseline and 1‐year study visit, and between the baseline and the 2‐year follow‐up visit. An *α* level <0.05 was defined as statistically significant.

## Results

### Clinical presentation and genetic distribution

A total of 99 patients (59 males) from 83 families met our inclusion criteria and were considered to have IPN. Nearly half of them (41/99) were descendants of adult patients who had a long‐term follow‐up at our institution,^12^ which enabled us to assess the pediatric patients at early stages of their disease. All held Spanish nationality except for one Chinese citizen, and the majority were Caucasian except for seven Romani descendants, two Afrodescendants, and one Asian. Of the 83 families, 78 (94%) were currently living in the Valencian Community. The average age at disease onset was 3.2 (±3.0), ranging from birth to 13.8 years of age, and the physical characteristics of each individual are described in Table 1. There were 85 patients from 72 families who presented with CMT (85.9% of the cohort), while 14 from 11 families had dHMN. Initially, 63 patients were classified as demyelinating CMT and 22 as axonal CMT. There were 11 patients that carried specific mutations and that did not undergo neurophysiological examination, and these individuals classified according to the index patient’s NCS results. These 11 patients were either CMT1A (*n* = 5), or they carried specific mutations in the *GDAP1* (*n* = 1), *GJB1* (*n* = 4), or *LITAF* (*n* = 1) genes. Regarding the pattern of inheritance in the entire cohort, this was considered as autosomal dominant (AD) in 55 cases (55.6%), 14 (14.1%) were autosomal recessive (AR), 9 (9.1%) were recessive X‐linked, 6 were de novo (6.0%), and 15 (15.2%) were considered sporadic. Consanguinity was detected in 7/99 cases (7.1%). A genetic diagnosis was achieved in 66 of the 83 families (79.5%), with a similar detection rate of mutations in the demyelinating (47/53; 88.7%) and the axonal (17/19; 89.5%) CMT families but significantly higher than in the dHMN families (2/11; 18.2%). Moreover, the genetic distribution in our cohort was compared with the latest published data on pediatric CMT (see Table 2).

**Table 1:** Physical description of children with inherited peripheral neuropathies.

Characteristic	Mean (SD) [Range]
Age when recruited, y	12.2 (4.3) [2 to 20]
Height, m	1.51 (0.20) [1.02 to 1.97]
Weight, kg	49.4 (19.6) [16.0 to 100.0]
BMI	20.6 (4.6) [12.8 to 32.2]
BMI percentile	58.3 (33.0) [0.0 to 99.0]
Foot posture index score	‐0.1 (3.5) [−12 to 7]
Ankle Lunge test, degrees	22.8 (16.8) [0.0 to 50.0]
CMTPedS total score at baseline	17.0 (9.2) [1 to 42]

The data included here correspond to both the CMT and dHMN phenotype: BMI, body mass index (calculated as weight in kilograms divided by height in meters squared); CMTPedS, Charcot–Marie–Tooth disease Pediatric Scale; CMT, Charcot–Marie–Tooth disease; dHMN, distal hereditary motor neuropathy.

**Table 2:** Genetic distribution and comparison to other pediatric series.

Gene	Number of patients (% of sample)			
	Present work (*n* = 99)	Cornett et al. (*n* = 520)	Hoebeke et al. (*n* = 75)	Fernandez‐Ramos et al. (*n* = 36)
*dupPMP22*	37 (37.4)	252 (48.5)	46 (61.3)	16 (44.4)
*GDAP1* AD	9 (9.1)			
*GDAP1* AR	1 (1.0)	3 (0.6)	1 (1.3)	
*GJB1*	8 (8.1)	10 (1.9)	2 (2.6)	1 (2.7)
*MFN2*	3 (3.0)	31 (6.0)	11 (14.7)	1 (2.7)
*MPZ*	3 (3.0)	15 (2.9)	1 (1.3)	
*HK1*	3 (3.0)		1 (1.3)	
*BICD2*	3 (3.0)			
*EGR2*	2 (2.0)			
*SH3TC2*	2 (2.0)	13 (2.5)		
*FGD4*	2 (2.0)	1 (0.2)		
*NDRG1*	1 (1.0)		1 (1.3)	1 (2.7)
*LITAF*	1 (1.0)	1 (0.2)		
*DYNC1H1*	1 (1.0)			
*ATP1A1*	1 (1.0)			
*ATL1*	1 (1.0)			
*ARSA*	1 (1.0)			
*PMP22point*		9 (1.7)		1 (2.7)
*TRPV4*		1 (0.2)		1 (2.7)
*GARS1*		4 (0.8)		
*NEFL*		3 (0.6)		
*MTMR2*		2 (0.4)		
*PRX*		1 (0.2)	1 (1.3)	
*FIG4*		4 (0.8)		
*CMTX3 locus*		6 (1.2)		
*HINT1*				2 (5.6)
*LMNA*			1 (1.3)	
*GAN*			1 (1.3)	
*YARS*			1 (1.3)	
*GDAP1* and *MFN2* ^1^			2 (2.6)	
Unidentified gene	20 (20.2)	127 (24.4)	6 (8.0)	13 (36.1)

Abbreviations: AD, autosomal dominant; AR, autosomal recessive; CMTX3 locus, large DNA interchromosomal insertion in Xq27.1.

^1^ Patients who carried combined heterozygous mutations in both *GDAP1* and *MFN2*;*dupPMP22*, CMT1A duplication; *PMP22point*, point mutation in the PMP22 gene.

### Patients with demyelinating CMT (CMT1)

Among these patients, the disease‐causing mutation was identified in 55 individuals with the following distribution: 37 carried a PMP22 duplication; 3 mutations in *GJB1*; 3 mutations in *MPZ*; 3 mutations in *HK1*; 2 mutations in *SH3TC2*; 2 mutations in *FGD4*; and one patient carrying mutations in *ARSA*, *LITAF*, *ATP1A1*, *EGR2,* or *NDRG1*. The patient with metachromatic leukodystrophy presented with a demyelinating polyneuropathy at the age of 14 months and a normal brain MRI. At 24 months of age, hyperintensities consistent with leukodystrophy were apparent in brain MRI. We consider the demyelinating neuropathy to be part of metabolic disorder. This patient carried two known mutations in the *ARSA* gene (NM_000487: [c.986C>T; p.Thr329Ile], [c.991G>T; p.Glu331*])^20^, ^21^ and an additional new variant classified as likely pathogenic according to the ACMG criteria (NM_000487: c.902G>C; p.Arg301Pro). We identified a new variant in the *ATP1A1* gene (NM_001160233: c.1645G>A; p.Gly549Arg) in an adolescent whose CMT was classified as intermediate (ulnaris MNCV 34.4 m/s, CMAP amplitude 12.3 mV). His affected father also carried the same variant and would have also been classified as having an intermediate form of CMT (ulnaris MNCV 41.3 m/s, CMAP amplitude 9.0 mV). Following ACMG criteria, the novel variant in *ATP1A1* was classified as likely pathogenic based on its absence in control databases (gnomAD, ExAC, 1000G), its co‐segregation with the disease in three generations of affected family members and a computationally predicted deleterious effect. We observed strong clinical heterogeneity between a proband and his father who both carried a heterozygous mutation in the *EGR2* gene (NM_000399: c.1142G>A; p.Arg381His). The son was diagnosed with CMT1 aged 4 (ulnaris MNCV <10 m/s), which spread to the upper limbs at 5, and he used a wheelchair and developed a diaphragmatic weakness at 7, dying at the age of 9 due to a bilateral pneumonia. His father was diagnosed at age 39 (ulnaris MNCV around 20 m/s) and his sole symptom since his early thirties had been high arched feet.

#### Patients with axonal CMT (CMT2)

**Figure 1:**
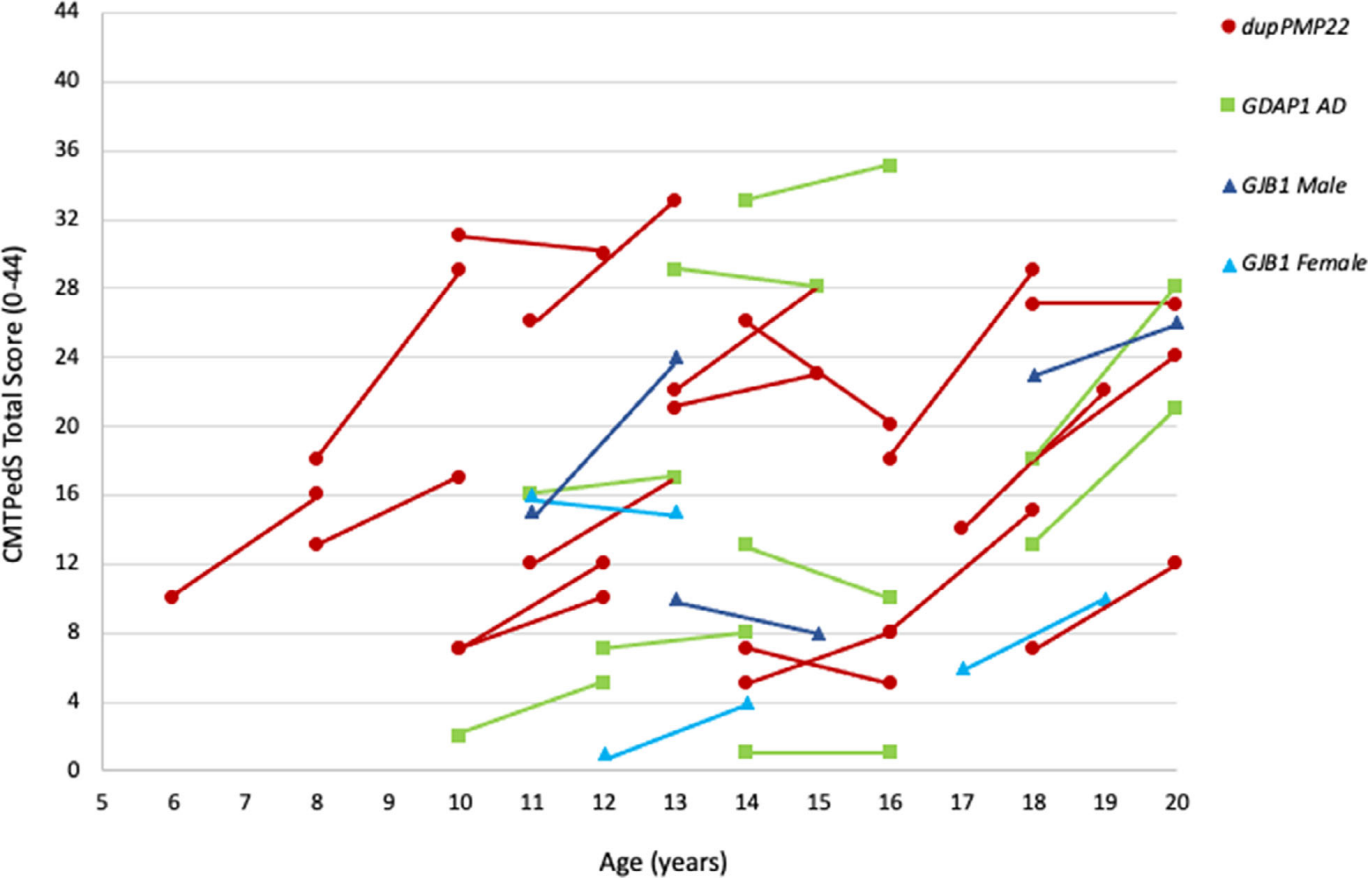
Progression of the total CMTPedS score in relation to the most common CMT genetic subtypes. Each slope represents a patient’s change in the CMTPedS Total score over 2 years. Abbreviations: CMTPedS, Charcot–Marie–Tooth disease Pediatric Scale (mild [0–14], moderate [15–29], and severe [30–44]); CMT, Charcot–Marie–Tooth disease; *dupPMP22*, CMT1A duplication; AD, autosomal dominant.

From a total of 22 patients with axonal CMT, we identified *GDAP1* mutations in 10, the most frequent cause of axonal CMT. Of these, nine patients harbored the AD *GDAP1* mutation (NM_018972: c.358C>T; p.Arg120Trp) and the remaining patient carried an homozygous mutation in *GDAP1* (NM_018972: c.844C>T; p.Arg282Cys). In terms of frequency, *GDAP1* was followed by various mutations in *GJB1* (*n* = 5) and *MFN2* (*n* = 3). Most carriers of the *GDAP1* p.Arg120Trp mutation (5/9) had a moderate CMTPedS score, despite the wide a range in clinical severity with two 12‐ and 13‐year‐old siblings scoring 29 and 33 in the CMTPedS, respectively (see Fig. 1). The only patient with a homozygous *GDAP1* mutation scored 22 in the CMTPedS at age 16 (within the moderate range). A heterozygous mutation in *EGR2* (NM_000399: c.1226G>A; p.Arg409Gln) and a de novo mutation in the *ATL1* gene (NM_015915: c.1223T>C; p.Met408Thr) were responsible for the disease in two other individuals. The one with the *ATL1* mutation presented with severe, infant onset axonal CMT, with spasticity and profound intellectual disability.

#### Patients with dHMN

From a total of 14 patients with dHMN, we identified disease‐causing variants in 5 of them: three siblings carried the c.320C>T (p.Ser107Leu) mutation in *BICD2* and one individual harbored a de novo mutation in *DYNC1H1* (c.917A>G; p.His306Arg). Detailed clinical and genetic descriptions on these set of patients have already been published.^22^

### Natural history

We assessed 76 children and adolescents with CMT using the CMTPedS, 62 of whom were also able to complete all 11 items of the scale 1‐year later and 45 of whom completed the CMTPedS over a 2‐year period. None underwent surgical correction between these assessments. The distribution of the genes affected in the CMT patients assessed with the CMTPedS was: PMP22dup (*n* = 32), *GDAP1* (*n* = 10), *GJB1* (*n* = 8), *MPZ* (*n* = 3), *HK1* (*n* = 3), *FGD4* (*n* = 2), *MFN2* (*n* = 2), *EGR2* (*n* = 2), *ATP1A1* (*n* = 1), *LITAF* (*n* = 1), *NDRG1* (*n* = 1), and an unidentified gene (*n* = 11).

The CMTPedS total score at baseline ranged from 1 (mild) to 42 (severe). There was significant progression over 1 year at a rate of 1.84 (±3.7) and over 2 years at a rate of 3.6 (±4.4) for all the genetic subtypes of CMT (*p* < 0.0005). Disease worsening was also significant for the most frequent genetic subtype, CMT1A (Table 3). The progression of the total CMTPedS score over 2 years is represented in figure 1 for the most common genotypes. There were insufficient numbers of children and adolescents to evaluate the disease progression associated with other genetic subtypes, such as CMT1B, CMT2A, and CMT4G. The most responsive items in a 1‐year period (*n* = 62) were grip strength (*z*‐score change of −0.6, 95% CI −1.0 to −0.25, *p* = 0.001) and long jump (*z*‐score change of −0.32, 95% CI −0.6 to −0.06, *p* = 0.017). This was also true for the 2‐year period (*n* = 45) with grip strength (*z*‐score change of −0.6, 95% CI −0.92 to −0.31, *p* < 0.0001) and long jump (*m*‐score change of −0.46, 95% CI −0.83 to −0.09, *p* = 0.017) showing a significant change relative to the baseline.

**Table 3:** Disability progression over 2 years according to the CMTPedS Total score in the most frequent CMT genetic subtypes.

CMT type	Baseline score [*n*]	1‐year FUP score [*n*]	2‐year FUP score [*n*]	Difference over a year	Difference over 2 years
All CMT cases	17.3 ± 9.7 (1–42) [76]	18.1 ± 10.1 (1–42) [62]	20.1 ± 10.1 (1–38) [45]	1.84 ± 3.7 (95% CI 0.89–2.79)**	3.6 ± 4.4 (95% CI 2.3–5.0)**
CMT1A	14.9 ± 7.0 (4–31) [33]	16.3 ± 7.8 (6–34) [29]	19.8 ± 8.3 (5–33) [19]	1.7 ± 3.6 (95% CI 0.33–3.1)*	4.2 ± 4.3 (95% CI 2.1–6.3)**
*GDAP1* AD	14.7 ± 11.0 (1–33) [9]	15.6 ± 10.9 (1–32) [9]	17.0 ± 11.8 (1–35) [9]	0.9 ± 3.3 (95% CI −1.6–3.4)	2.3 ± 4.2 (95% CI −0.9–5.5)
*GJB1*	12.8 ± 6.8 (1–23) [8]	14.8 ± 8.3 (2–23) [6]	14.5 ± 8.9 (4–26) [6]	3.0 ± 4.0 (95% CI −1.2–7.2)	2.7 ± 3.9 (95% CI −1.5–6.8)

The data are the mean ± SD (range) for baseline, and the 1 and 2 year follow‐up scores, and the mean ± SD (95% Confidence Interval) for the differences: **Significant change from baseline (*p* < 0.0005); *Significant change from baseline (*p* < 0.05). Abbreviations: CMTPedS, Charcot–Marie–Tooth disease Pediatric Scale; CMT, Charcot–Marie–Tooth disease; FUP, follow‐up; AD, autosomal dominant.

## Discussion

In the present series of IPN patients, 80.6% of the individuals obtained a genetic diagnosis (*n* = 99), a very similar value to the 83.3% described in the adult series from the same Western Mediterranean area (*n* = 438)^12^ and close to the 75% observed in a large pediatric series reported by the International INC (*n* = 520).^5^ The success rates for genetic diagnosis in CMT1 and CMT2 families were comparable in our cohort (88.7% and 89.5%, respectively), although other studies carried out mainly on adults reported higher genetic hit rates for CMT1 than CMT2 (approximately 90–95% vs. 40–60%).^2^, ^11^, ^12^ Our high rate of mutation detection among CMT2 patients may be related to the large proportion of patients carrying heterozygous mutations in the *GDAP1* and *GJB1* genes. We found CMT1A to be the most common genetic subtype of CMT, in accordance with most pediatric series.^5^, ^8^, ^9^ Our second most frequent subtype was that carrying AD mutations in the *GDAP1* gene. This genetic subtype was not present in other pediatric cohorts^5^, ^8^, ^9^ and the high prevalence of AD *GDAP1* mutations in our series most likely reflects a founder effect of this specific mutation: p.Arg120Trp.^23^ The two most severely affected patients with dominant *GDAP1* mutations were children of a clinically asymptomatic father. Subsequent studies showed that they also carried a mutation in the *JPH1* gene inherited from their healthy mother.^24^ JPH1 and GDAP1 play a role in calcium homeostasis, and the combination *JPH1* p.Arg213Pro and *GDAP1* p.Arg120Trp leads to a reduced store‐operated calcium entry activity.^24^ The patient in our cohort with the homozygous p.Arg282Cys mutation in the *GDAP1* gene had a milder phenotype than the majority of patients harboring AR inherited mutations in this gene.^25^ It is interesting to note that the only family described with the same mutation also had a fairly benign course.^26^ Our cohort had few cases of *MFN2* neuropathy (3%), like other Spanish series,^9^, ^12^ yet this contrasted with other pediatric cohorts from the INC^5^ and in France,^8^ in which *MFN2* mutations were the second most frequent.

Here, we described a new pathogenic variant in the *ATP1A1* gene and its clinical correlations. Mutations in *ATP1A1*, which encodes the alpha‐1 subunit of the Na^2+^/K^+^‐ATPase, were recently identified as a cause of AD axonal CMT in seven unrelated families.^27^ Subsequently, heterozygous de novo mutations in *ATP1A1* were reported in individuals with renal hypomagnesemia, refractory seizures, and intellectual disability,^28^ and in a child with spastic paraplegia and intellectual disability with normal nerve conduction.^29^ Our patient with the new AD variant in the *ATP1A1* gene (p.Gly549Arg) had no pyramidal signs or intellectual disability at examination, and his CMT could be classified as intermediate, in accordance with previous observations from two Chinese families.^30^ The child carrying the p.Arg381His variant in the *EGR2* gene suffered from a very severe demyelinating phenotype but did not show signs of cranial neuropathy. This same *EGR2* mutation was previously associated with severe demyelinating CMT with cranial nerve involvement.^31^ The severe disability displayed by our proband carrying the p.Arg381His *EGR2* mutation contrasted with the mild presentation of his father. This might suggest that this mutation is not the sole alteration responsible for the individual’s phenotype and that genetic modifiers may influence the clinical heterogeneity.^32^ In patients with dHMN, mutations were only identified in genes like *BICD2* and *DYNC1H1* that are responsible for lower extremity dominant spinal muscular atrophy (SMALED). Our patients fit this phenotype. Indeed, we did not find any individual’s carrying mutations in *HSPB1*, even though this is a gene very frequently affected in dHMN patients, confirming the late onset of mutations in this gene.^33^

Regarding clinical outcome measures of CMT, CMTPedS reliably measures disability in children and adolescents from 3 to 20 years of age, and it can detect change over 2 years.^7^ Multicenter natural history data (*n* = 187) showed significant disease progression over 2 years at a rate of 2.4 (±4.9) for all genetic subtypes of CMT (*p* < 0.001) and at a rate of 1.8 (±4.2) for CMT1A (*p* < 0.001).^34^ We also found the scale to be sensitive to change over 2 years in our single‐center cohort. However, the rate of progression was higher in our CMT1A subgroup (4.2 ± 4.3, *p* < 0.0005, *n* = 19) than in our overall CMT cohort (3.6 ± 4.4, *p* < 0.0005, *n* = 45), which might reflect the effect of the slower progression in patients with AD *GDAP1* mutations (2.3 ± 4.2, *n* = 9) as these latter patients were 20% of the cohort at the 2‐year follow‐up. CMT caused by the AD p.Arg120Trp mutation in the *GDAP1* gene is thought to be particularly mild and with slow progression.^23^ The progression of CMT1A, AD *GDAP1,* and *GJB1* was fairly variable over 2 years in each individual, perhaps influenced by growth as suggested previously.^5^ Our results support the capacity of CMTPedS to detect disease progression over 1 year, all CMT and CMT1A patients displaying significant progression. Foot dorsiflexion strength, balance and the length of jumping were previously found to be the most responsive items.^34^ In our cohort, the most responsive items were grip strength and the length of jumping. To optimize CMT clinical trials, the INC estimated that young (3–8 years) and mildly affected (CMTPedS score <15) CMT1A cases were the most responsive in terms of the CMTPedS over 2 years of disease progression.^35^ The observation that the CMTPedS can also detect significant progression over 1 year and that grip strength is also a responsive item may help further optimize the design of forthcoming clinical trials.

Our prospective study is not without limitations. Most genetic subtypes had three patients or less, precluding a natural history analysis. As these patients were recruited from a neuromuscular clinic, patients in whom peripheral neuropathy was not the cardinal feature may be underrepresented.

## Conclusions

This large pediatric series of IPN from a single tertiary center highlights the distinctive genetic distribution in this Mediterranean region, with more AD *GDAP1* patients than *MFN2*. Evaluation in children and adolescents may be key to highlight the role of modifier genes (e.g., JPH1 as a modifier of GDAP1‐associated CMT), since at their young age they have not been exposed to external factors like medical co‐morbidities, drugs or other toxins. This study also shows the CMTPedS to be sensitive to disease change over 1 year, which may help design more efficient therapeutic trials in children of any rational therapy that aims to slow or halt the progression of CMT.

## Author Contributions

HAE and TS designed the study. HAE assessed the patients, conducted the statistical analysis, and elaborated the first draft. MF, JFVC, EMS, IP, MT, and TS provided detailed patient information and critically revised the manuscript. CE and VL performed the genetic studies and reviewed the article for important intellectual content.

## Conflict of Interest

All authors read and approved the final manuscript and have no conflicts of interest to disclose.

## Ethics Approval and Consent to Participate

Written informed consent was obtained from all the patients or their guardians/parents, and the protocols were approved by the Institutional Review Board and Ethics Committee at the Hospital Universitari i Politècnic La Fe (Valencia, Spain).

## Patient Consent for Publication

Not applicable.

## Data Availability

The data that support the findings of this study are available from the corresponding author upon reasonable request.
